# Universal CARs, universal T cells, and universal CAR T cells

**DOI:** 10.1186/s13045-018-0677-2

**Published:** 2018-11-27

**Authors:** Juanjuan Zhao, Quande Lin, Yongping Song, Delong Liu

**Affiliations:** The Affiliated Cancer Hospital of Zhengzhou University and Henan Cancer Hospital, 127 Dongming Road, Zhengzhou, 450008 China

## Abstract

Currently, the two approved T cell products with chimeric antigen receptors (CAR) are from autologous T cells. These CAR T cells approved for clinical use must be generated on a custom-made basis. This case-by-case autologous T cell production platform remains a significant limiting factor for large-scale clinical application due to the costly and lengthy production process. There is also an inherent risk of production failure. The individualized, custom-made autologous CAR T cell production process also posts constriction on the wide application on diverse tumor types. Therefore, universal allogeneic T cells are needed for the preparation of universal CAR T cells that can serve as the “off-the-shelf” ready-to-use therapeutic agents for large-scale clinical applications. Genome-editing technologies including ZFN (zinc finger nuclease), TALEN (transcription activator-like effector nuclease), and CRISPR-Cas9 are being used to generate the universal third-party T cells. In addition, split, universal, and programmable (SUPRA) CARs are being developed to enhance the flexibility and controllability of CAR T cells. The engineered universal T cells and universal CARs are paving the road for a totally new generation of CAR T cells capable of targeting multiple antigens and/ or being delivered to multiple recipients without re-editing of T cells. This may escalate to a new wave of revolution in cancer immunotherapy. This review summarized the latest advances on designs and development of universal CARs, universal T cells, and clinical application of universal CAR T cells.

## Introduction

**Fig. 1:**
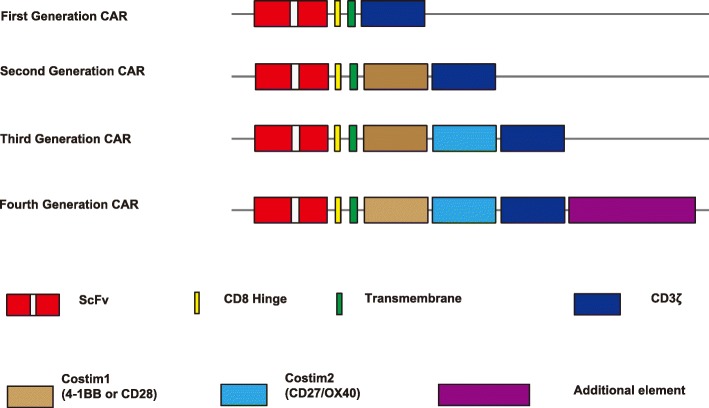
Structures of chimeric antigen receptors (CAR). First generation of CARs contains the single chain variable region (scFv) of a monoclonal antibody, T cell receptor transmembrane domain, and an intracellular signaling domain of CD3 zeta chain. The second generation of CARs contains a single co-stimulatory domain (CD28 or 4-1BB), whereas the third generation of CARs may have two or more co-stimulatory domains (CD27, CD28, 4-1BB or OX40). The fourth generation CARs contain a controllable on-off switch or a molecule (additional element) to enhance T cell function, enrichment, and minimize senescence

Chimeric antigen receptors (CARs) are engineered receptors that typically contain the antigen-binding region of a monoclonal antibody (mAb), T cell receptor transmembrane domain, and an intracellular signaling domain of CD3 zeta chain [1–7]. This is the structure of the first generation of CARs (Fig. 1) [8, 9]. Upon binding to a specific antigen, CAR can transmit the signal and activate the T cells. The T cells that have been genetically engineered to express CAR can undergo specific immune responses, avoiding the restriction traditionally conferred by the major histocompatibility complex (MHC).

The first generation of CAR T cells was found to have limited proliferative capacity and short survival. Currently, the FDA-approved CAR T cell products belong to the second generation of CAR T cells [9, 10]. The second generation of CARs contains a single co-stimulatory domain (CD28 or 4-1BB), whereas the third generation of CARs may have two or more co-stimulatory domains (CD27, CD28, 4-1BB, or OX40) [9, 11–18] (Fig. 1). Additional molecular elements have been inserted into the CAR constructs to express functional transgenic proteins [10, 19–22]. This defines the fourth generation CARs which may contain a controllable on-off switch, a suicide gene, or a molecule to enhance T cell function, enrichment, and minimize senescence [21, 23]. Over the last few years, significant modifications have been made to further improve the CAR T designs. Bispecific CARs can simultaneously target two antigens and/or epitopes to limit immune escape [24]. Universal CARs are being developed to increase flexibility and controllability as well as scalability. To increase efficacy and potency, functional elements such as interleukin genes are inserted into the fourth generation CAR constructs. To increase safety and controllability, on-off-switches or suicide genes are built into the new CARs.

The enormous potential of the CAR T cells has been confirmed in clinical studies of adult and pediatric cancer treatment [7, 13, 25–29]. Two CD19-engineered CAR T cell products have been approved for clinical treatment of B lymphoid malignancies [27, 30–38]. These CAR T cells are autologous lymphocytes from patients. However, this patient-specific autologous T cell paradigm is a significant limiting factor for large-scale deployment of the CAR technology as the CAR T cell product is individualized and therefore varies from patient to patient. It is hence not a ready-to-use preparation of conventional therapeutic agents. The individualized manufacturing process is costly and time-consuming. In addition, generation of sufficient number of custom-made autologous T cells may not be feasible or successful in all cases, particularly for infants or highly treated patients who are profoundly lymphopenic owing to multiple previous chemotherapies and/or stem cell transplantation. Furthermore, each CAR has a fixed antigen specificity such that each CAR T preparation can only target one epitope of a specific antigen, limiting the efficacy due to heterogeneous tumor antigen expression and tumor antigen escape. The universal “off-the-shelf” CAR T cells that can be simultaneously or sequentially administered to multiple patients can effectively solve the above problems. This review summarized the recent advances in the designs and applications of universal CAR T cells.

### Universal CARs: design principles and early studies

The current CAR T cell therapy is limited by antigen specificity and scalability since each CAR T cell system targets only one antigen or two antigens [24]. To increase flexibility and expand antigen recognition, new CARs are being designed and tested as universal CARs. These CARs use a “third-party” intermediate system that splits the antigen-targeting domain and the T cell signaling unit [39–41]. This “third-party” “lock-key” split CAR system confers CAR T cells with near-infinite antigen specificity.

#### BBIR CAR: biotin-binding immune receptor

**Fig. 2:**
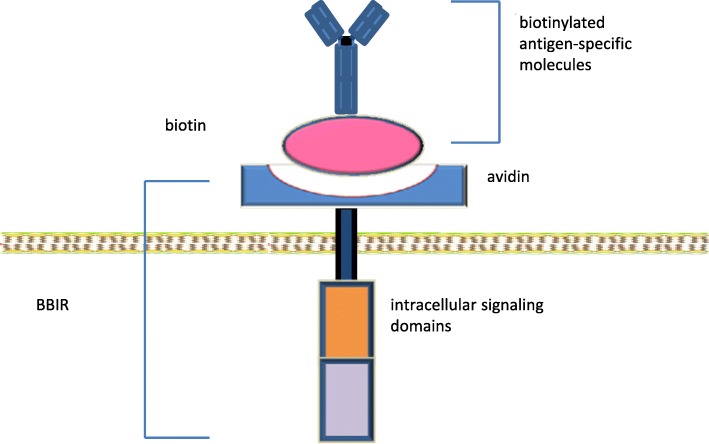
The structure of BBIR CAR. In the BBIR CAR, avidin serves as the extracellular ligand binding domain and is linked to the transmembrane and intracellular signaling domains. The biotinylated antigen-specific molecules can be full-length antibodies, scFvs, or other tumor-specific ligands. Through the binding of biotin to avidin, any extracellular signal linked to the biotin can activate the T cells. Therefore, the BBIR CAR remains constant and can serve as a universal CAR. (adapted from Urbanska, K, et al.; *Cancer Res*. 2012; 72(7); 1844–1852)

To use the biotin-avidin “lock-key” mechanism for CAR engineering, avidin was used as the extracellular domain linked to an intracellular T cell signaling domain (Fig. 2) [39]. This creates a system of biotin-binding immune receptors (BBIR). The BBIR containing dimeric avidin (dcAv BBIR) was able to efficiently recognize and bind a variety of biotinylated antigen-specific molecules (scFV, mAbs, or tumor-specific ligands), whereas the BBIR containing monomeric avidin (mcAv BBIR) did not result in a specific immune response, probably due to poor affinity between biotin and monomeric avidin.

dcAv BBIRs were able to react specifically to targeted antigens in the presence of extremely low concentrations of biotinylated molecules (such as Bio-EpCAM Ab at 0.1 ng/mL). The same batch of genetically modified T cells was shown to recognize multiple tumor-associated antigens (TAAs), FRα, EpCAM, and mesothelin, respectively, after the addition of matched biotinylated antibodies (Abs). The BBIRs can also sequentially recognize diverse TAAs via stepwise addition of corresponding biotinylated Abs [39]. These findings confirmed the versatility of the BBIR platform.

The BBIR T cells showed a strong inhibition of tumor growth in the xenograft mouse model of human ovarian cancer following the addition of biotinylated corresponding antibody [39]. This BBIR platform of universal CAR system has the potential to significantly extend conventional CAR approaches and generate T cells of unlimited antigen specificity.

Using the biotin-avidin system, a similar universal CAR was constructed [40]. The anti-tag CAR T cells were recently tested to target CD 19^+^ and CD 20^+^ cells. One concern of this system is the immunogenicity of avidin. Further testing of the biotin-avidin anti-tag CAR T cells in an animal model and clinical trial will be needed.

#### SUPRA CARs

**Fig. 3:**
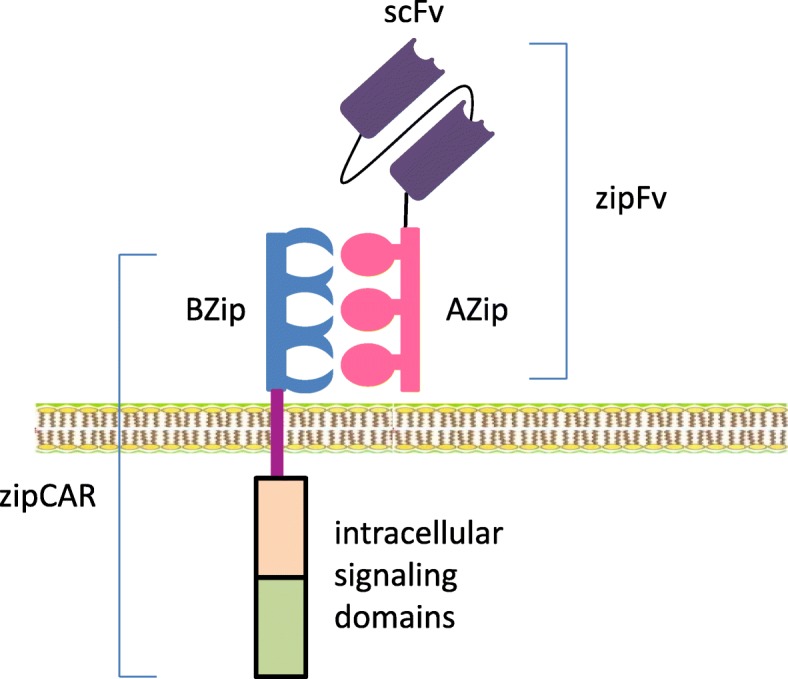
The structure of SUPRA CAR. A SUPRA CAR system consists of a zipCAR and a zipFv. A zipFv has a scFv linked to a leucine zipper (AZip). A zipCAR has a cognate leucine zipper (BZip) that can bind to the AZip. Through the binding between A- and B-leucine zippers, any extracellular signal linked to the AZip can activate the T cells. Therefore, the SUPRA CAR remains constant and can serve as a universal CAR. The affinity between the A- and B-leucine zippers can be adjusted so that the signaling strength and activity can be dialed up or down as desired. When AZip is linked to a null antigen, the signaling is quenched, and T cells become inactive. (adapted from Choi, JH, et al., *Cell* 2018; 173: 1–13)

To enhance the flexibility and controllability of CARs, a split, universal, and programmable (SUPRA) CAR system was invented [42]. SUPRA CAR is a two-component receptor system consisting of a universal receptor with leucine zipper adaptor (zipCAR) on T cells and a separate scFv with leucine zipper adaptor (zipFv) molecule targeting specific antigens (Fig. 3). This split “lock-key” structure of SUPRA CAR with leucine zipper adaptors indeed has several clinically relevant advantages over a rigid CAR system:

1. Antigen targets of one set of zipCAR T cells can be switched, expanded and combined without further modification of the CAR T cells. Targets of the split scFv adaptor can be switched easily without affecting the effector T cells since the zipCAR T cells remain unchanged. This therefore expands the repertoire of targetable antigens for a set zipCAR T system. This theater-array combination of different targets of effector T cells can effectively counter tumor escape, and reduce relapse.
2. The activities and toxicities of SUPRA CAR T cells can be controlled and fine-tuned. This can be achieved by adjusting the binding strength through variable leucine zipper configurations. This leads to the defined potency of T cell signaling and thus the degree of T cell activation. By the same mechanism, a zipFv with no specific antigen target can be used to competitively tune down zipCAR T cell activation to minimize or terminate the toxicities.

3. Signaling domains and effector cell types can be changed and combined. Orthogonal zipCARs can be connected to different intracellular signal domains in the same cell or assembled to differently engineered cell types with defined ratios, thus enabling precise controllability of different signal domains or cells types (such as NK cells), potentially achieving the precise, tailored fine-tuning of effector cell responses [42].

In the mouse xenograft models of breast cancer and leukemia, the SUPRA CAR system demonstrated a robust anti-tumor effect comparable to that of conventional CAR T cells [42]. With the versatility and controllability, the SUPRA CAR system represents a superior universal CAR design.

### Universal T cells: design principles and preclinical studies

#### Generation of universal T cells

Currently, the two approved CAR T cell products are from autologous T cells. These CAR T cells approved for clinical use must be generated on a custom-made basis.

This patient-specific autologous T cell production platform calls for highly skilled and dedicated facility as well as substantial investment in infrastructure. In addition, it is very time-consuming and precludes immediate administration when the most critically ill patients need the therapy, since the generation of a therapeutic product requires a lengthy and individualized manufacturing process. There is also an inherent risk of production failure as there is no guarantee that the generation of sufficient number of custom-made autologous T cells can always be feasible for patients who are critically ill and profoundly lymphopenic owing to previous chemotherapy, radiation therapy, and/or stem cell transplantation. This costly and time-consuming process also posts constriction on the wide application on diverse tumor types. Therefore, universal T cells are needed for the preparation of universal CAR T cells.

The main design principle for generating universal CAR T cells is to generate tumor antigen-specific T cells from allogeneic healthy donors. The common basis of this method is to effectively abolish graft-versus-host disease (GVHD) by genetically disrupting the TCR gene and/or HLA class I loci of the allogeneic T cells. By targeting genomic sequences in the constant regions of the endogenous α or β subunits of the TCR or disrupting HLA-A locus of MHC gene complex, the expression of TCR or the HLA class I antigens is abrogated, and the resulting T cells are not capable of recognizing allogeneic antigens, thus leading to the elimination of GVHD. These allogeneic T cells from healthy donors are universal T cells and can be used to generate universal CAR T cells for specific antigens of interest. Zinc finger nuclease (*ZFN*)[43, 44], transcription activator-like effector nuclease (TALEN)[45], and CRISPR/Cas9 system [23, 46, 47] are the most commonly used gene-editing methodologies for generating TCR-deficient and HLA class I-deficient T cells.

#### ZFN: zinc finger nuclease

**Fig. 4:**
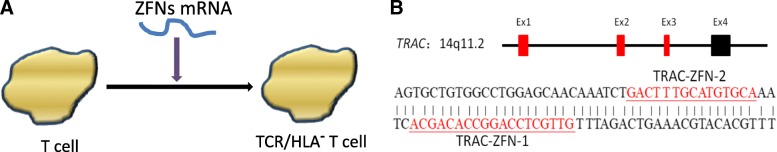
Production of universal TCR^−^/HLA^−^ T cells using ZFN (zinc finger nuclease). **a** The T cells were obtained from healthy adult donors. ZFN mRNA pairs were delivered to the T cells by electroporation. ZFN pairs designed to target TCR αor βconstant regions and HLA-A lead to DNA double-strand breaks at the given sites and cause deletion or insertion of nucleotides, resulting in permanent loss of gene expression. These TCR^−^/HLA^−^ T cells made from allogeneic healthy donors can be used as universal T cells for preparation of “off-the-shelf” CAR T cells. **b** Structure of the ZFN pair. One ZFN pair was designed to bind exon 1 of the TCRα constant region (TRAC), and the underlined nucleotide sequences represent the targeted binding sequences. The red blocks represent coding regions and the black block represents a noncoding region (adapted from Torikai H, et al. *Blood* 2012; 119(24):5697–5705)

ZFN is a designed specific DNA endonuclease that can result in a double-strand break of a user-defined gene target. Repairing the cleaved sites through non-homologous end joining or homology-directed DNA repair pathway can cause deletion or insertion of nucleotides, resulting in permanent loss of the target gene expression [48–51]. Using this genome-editing technology, the expression of TCRα constant (TRAC) or the TRBC chains in T cells were disrupted, leading to loss of TCR function [43, 44]. These modified T cells were shown to be anergic and not able to respond to TCR-specific stimulation. These CAR T cells were also shown to have no imbalance of T cell subsets (CD4^+^ or CD8^+^) [43]. Using the same approach, the HLA-A gene was disrupted. This population of ZFN-edited T cells was readily enriched through negative selection for HLA-positive cells [44]. These TCR-deficient or HLA-A-deficient T cells did not cause GVHD in animal models. The TCR^neg^ T cells have been used for generation of universal CAR T cells which may offer an “off-the-shelf” immunopharmaceuticals [43, 44] (Fig. 4). Clearly, further clinical testing is needed to confirm the clinical potential.

#### TALEN: transcription activator-like effector nuclease

**Fig. 5:**
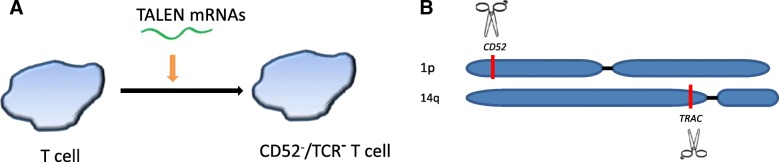
Production of universal CD52^−^/TCR^−^ T cells using TALEN (transcription activator-like effector nuclease). **a** The T cells were obtained from healthy adult donors. TALEN mRNAs targeting specific sites of *CD52* and the constant region of the T cell receptor α chain (*TRAC*) were electro-transferred into the T cells at the same time leading to permanent loss of gene expression. These CD52^−^/TCR^−^ T cells can be used for the preparation of “off-the-shelf” CAR T cells. **b** Diagram of TALEN-targeted sites. The blue band represents the chromosome. Scissors represent TALENs. The red blocks represent the targeted sites of TALENs. (adapted from Poirot, L, et al., *Cancer Res*. 2015; 75(18); 3853–3864)

Transcription activator-like effector (TALE) was initially discovered in plant [52, 53], and TALE nuclease (TALEN) is another designed site-specific endonuclease that has been used for genome-editing in a variety of species [54–57]. By application of TALEN gene-editing technology, the TCRα constant (TRAC) gene was disrupted, eliminating the potential for T cells to react to allogeneic antigens and mediate GVHD [45]. To abolish the possibility of any remaining alloreactive T cells and facilitate the engraftment of third-party CAR T cells, CD52 gene in the CAR T cells was simultaneously disrupted by TALEN (Fig. 5) [45]. This established a process for the large-scale manufacturing of healthy readily available “off-the-shelf” T cells deficient in expression of both T cell receptor and CD52. Alemtuzumab, a monoclonal antibody against CD52, has been used to destroy and eliminate remaining CD52^+^ wild-type alloreactive T cells, thereby enriching the desired TCR-less CAR T cells. These TALEN-edited healthy allogeneic T cells do not incite GVHD and are resistant to destruction by alemtuzumab. These T cells were used for the generation of universal CAR T cells.

As a proof of concept for the general application of this platform, TCR/CD52-deficient CD19 CAR T cells (dKO-CART19) were generated [45]. The dKO-CART19 universal T cells were shown to be deficient of alloreactivity, and the engraftment was facilitated by alemtuzumab. These universal T cells demonstrated antitumor activity indistinguishable from standard CD19 CAR T cells [45]. This manufacturing platform therefore has the potential for large-scale manufacturing of universal CAR T cells that are similar to a traditional drug ready for administration as an “off-the-shelf” agent.

#### CRISPR/Cas9: clustered regularly interspaced short palindromic repeat (CRISPR)/ CRISPR- associated protein 9 (Cas9)

**Fig. 6:**
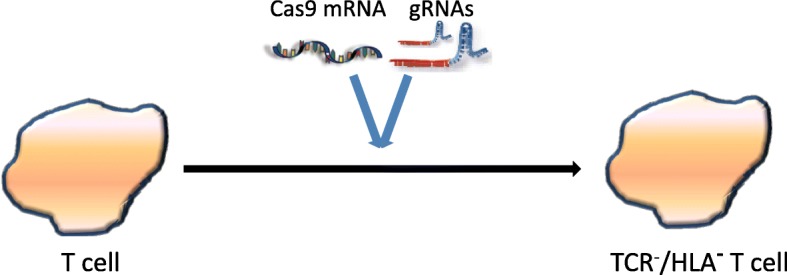
Production of universal TCR^−^/HLA^−^ T cells using the CRISPR/Cas9 system (clustered regularly interspaced short palindromic repeat /CRISPR- associated protein 9). The T cells were obtained from healthy adult donors. Cas9 mRNA and gRNAs targeting the constant region of the T cell receptor αor βchain (*TRAC* or *TRBC*) and *B*_*2*_*M* were transferred to T cells by electroporation. The Cas9 endonuclease is capable of cleaving the *TRAC*, *TRBC*, and *B*_*2*_*M* under the guidance of corresponding gRNAs and inducing indels at pre-validated sites. These universal TCR^−^/HLA^−^ T cells with reduced alloreactivity can be used for the preparation of “off-the-shelf” CAR T cells. (adapted from Ren, J, et al. *Clin Cancer Res*. 2016; 23(9); 2255–2266)

Both ZFN- and TALEN-based methodologies require designing specific pairs of nucleases for each new gene target. This has a great limit on the wide application of the technology. The CRISPR-Cas9 system was discovered in 2012 [58]. CRISPR-Cas9 system has been widely used for genome editing since it can be efficiently programmed to cleave DNA and induce indels at sites of interest [59–67]. Highly efficient double knockout of endogenous TCR and HLA class I (b2-microglobulin, B2M) was achieved via a one-shot CRISPR protocol to generate allogeneic universal CAR T cells (Fig. 6) [23, 68]. With this technology, multiplex genome editing was possible. Additional genes, such as PD1 and CD52 were edited, and universal CAR T cells were generated and proven to be functional as designed.

Through a CRISPR-CAR coupled lentiviral vector, CAR19 was introduced simultaneously, leading to the production of TCR-depleted CAR19 T cells [46]. The universal CAR19 T cells were highly homogeneous and had potent antileukemia activity in a human: murine chimeric tumor model. This approach was shown to be scalable through an automated separation process and may serve as a good platform for the generation of “universal” human T cells on a clinical scale.

CRISPR-Cas9 was used successfully for multiplex gene editing in CAR T cells [69, 70]. Two (TRAC and B2M) or three genes (TRAC, B2M, and PD-1) were disrupted in the CAR T cells [69]. TRAC and B2M were disrupted with high efficiency, yet only 64.7% of the clones of the PD-1 PCR products were mutants. This implies that T cells with PD-1 mutations were not optimally enriched by the negative selection-based enrichment method as the PD-1 expression was downregulated during T cell expansion. The TCR-deficient CAR T cells were shown to have cytotoxic anti-tumor functions in vitro and in vivo. The safety and efficacy of these TCR^neg^ CAR T cells must be characterized in clinical trials.

### FT819, iPSC CAR iT cells

A universal CAR 19 T cell line, FT819, was generated from an induced pluripotent stem cell (iPSC) line [71]. An iPSC cell line was established from healthy peripheral blood T cells which also contain a CD19-targeted CAR and bi-allelic TRAC locus disruption. TCR-less CAR19 T cells were successfully generated from the iPSC master line. The CAR19 iT cells were fully functional in vitro. A clinical trial in human is required to fully assess the safety and efficacy of this first-of-the-kind off-the-shelf CAR iT cell product.

### Clinical studies of universal CAR T cells

TALEN gene-edited TCR-deficient CAR T cells are being studied in humans through clinical trials. Two infants with highly relapsed refractory CD19^+^ B-ALL were treated with the allogeneic universal CAR T cells [72]. The two children achieved cytogenetic and molecular remission after the universal CAR T therapy and went on to receive allogeneic stem cell transplantation successfully.

Clinical trials of CRISPR/Cas9 gene-edited universal allogeneic CAR T cells have been initiated (NCT03166878, NCT03229876). Both were CD19-targeted CAR T cells. Details and outcomes are not yet available.

### Future perspectives

By disrupting TCR expression and/or MHC on allogeneic third-party T cells, universal T cells have been generated. These T cells do not cause GVHD and can be widely used to generate “off-the-shelf” CAR T cells. However, there are still key issues that urgently need to be resolved before the practical application of universal CAR T cells. For example, there exists the risk of GVHD caused by even less than 1% of TCR^+^ cells remaining in TCR^−^ cell preparation. The potential off-target effects and abnormal genotypes that arise during gene editing still require attention and further researches [73, 74]. Universal CAR T cell therapy is still at its infancy. Many issues require further explorations, particularly in the following areas:

1. Clinical trials in more patients to ascertain the efficacy and toxicity
2. Long-term follow-up to monitor the rate of acute and chronic GVHD, rejection, T cell exhaustion/senescence, and target antigen escape
3. Combination of allogeneic CAR T cells either sequentially and/or simultaneously to target multiple antigens in one type of cancer
4. Establishment and unification of clinical, industrial, and regulatory standards for universal CAR T cell therapy, such as the criteria for efficacies and toxicities, and manufacturing standards throughout the world
5. Revision and adoption of new reimbursement models for universal CAR T cell therapy since this type of therapy has high upfront cost and may induce long-term remission/cure or turn cancer into a chronic illness through repeated or maintenance infusions of CAR T cells
6. Application of CAR T therapy for other illnesses like autoimmune diseases and AIDS

## Conclusion

Genome-editing methodologies including ZFN, TALEN, and CRSIPR-Cas9 make it possible to generate universal third-party T cells. Split, universal, and programmable (SUPRA) CARs offer the flexibility and controllability of CAR T cells. A new generation of universal CAR T cells is in clinical trials and offers novel armamentarium for cancer immunotherapy.

## Abbreviations

CAR: Chimeric antigen receptor
CRISPR/Cas9: Clustered regularly interspaced short palindromic repeat (CRISPR)–CRISPR- associated 9 (Cas9)
TALEN: Transcription activator-like effector nuclease
ZFN: Zinc finger nuclease
